# Simulating the methodological bias in the ATLS classification of hypovolemic shock: a critical reappraisal of the base deficit renaissance

**DOI:** 10.1186/s13049-024-01276-0

**Published:** 2024-10-25

**Authors:** M. L. A. Heldeweg, T. T. R. Heldeweg, J. A. H. Stohlmann, P. Freire Jorge, R. Boer, L. A. Schwarte, P. Schober

**Affiliations:** 1 Department of Anesthesiology, Amsterdam University Medical Centers, Vrije Universiteit Amsterdam, Location VUmc, Amsterdam, The Netherlands; 2 Master of Science in Engineering, Enschede, The Netherlands; 3 Department of Intensive Care Medicine, University Medical Center Groningen, Groningen, The Netherlands; 4 Department of Anaesthesia and Intensive Care Medicine, Third Faculty of Medicine, Charles University, FNKV University Hospital, Prague, Czech Republic

**Keywords:** Advanced trauma life support, Base deficit, Base excess, Heart rate, Systolic blood pressure, Glasgow coma scale, Transfusion, Methodology, Bias, Simulation

## Abstract

**Background:**

The Advanced Trauma Life Support classification (ATLS) of hypovolemic shock is a widely used teaching and treatment reference in emergency medicine, but oversimplifies clinical reality. A decade ago, a landmark study compared vital parameters to base deficit (BD) in trauma patients. The investigators concluded that BD had higher accuracy to detect the need for early blood product administration. BD was subsequently introduced in the ATLS shock classification and has since been widely accepted as a laboratory standard for hypovolemia. The aim of this study is to investigate whether a methodological bias may have inadvertently contributed to the study’s results and interpretation.

**Methods:**

In the current study, we replicate the original study by simulating a cohort of trauma patients with randomly generated data and applying the same methodological strategies. First, a predefined correlation between all predictor variables (vital parameters and BD) and outcome variable (transfusion) was set at 0.55. Then, in accordance with the methods of the original study we created a composite of ATLS parameters (highest class amongst heart rate, systolic blood pressure, and Glasgow Coma Scale) and compared it with BD for resulting transfusion quantity. Given the preset correlations between predictors and outcome, no predictor should exhibit a stronger association unless influenced by methodological bias.

**Results:**

Applying the original imbalanced grouping and composite allocation strategies caused a systematic overestimation of shock class for traditional ATLS parameters, favoring the association between BD and transfusion. This effect persisted when the correlation between BD and transfusion was set substantially worse (rho = 0.3) than the correlation between ATLS parameters and transfusion (rho = 0.8).

**Conclusions:**

In this fully reproducible simulation, we confirm the inadvertent presence of methodological bias. It is physiologically reasonable to include a metabolic parameter to classify hypovolemic shock, but more evidence is needed to support widespread and preferred use of BD.

**Supplementary Information:**

The online version contains supplementary material available at 10.1186/s13049-024-01276-0.

## Background

The Advanced Trauma Life Support (ATLS) shock classification is an international clinical reference standard and teaching instrument for hypovolemic shock severity [1]. Traditionally, the classification was based on a combination of vital signs including heart rate (HR), systolic blood pressure (SBP), and Glasgow Coma Scale (GCS). However, only 9.3% of trauma patients are in a single shock class based on their vital signs [2]. In an effort to improve and expand the ATLS classification, Mutschler and colleagues compared base deficit (BD) to traditional ATLS parameters. BD is a surrogate marker of metabolic acid–base status and may therefore reflect tissue hypoxemia in hypovolemic shock. Although previous studies described BD’s use as prognosticator and measure of resuscitative effort, this was the largest comparison with traditional vital parameters. In a landmark publication, the investigators concluded that BD may be superior for identification of transfusion requirements [3]. Consequently, BD was incorporated into the updated ATLS classification of hypovolemic shock.

A decade later, BD has been widely embraced as a singular laboratory standard for assessment of acute hypovolemia, permeating clinical practice in emergency medicine and critical care. At least 59% of surveyed anesthesia and critical care clinicians use BD to guide (intraoperative) fluid management [4]. This is an important development, as a point-of-care laboratory parameter is undoubtedly a valuable addition to a clinician’s diagnostic toolkit. However, BD’s widespread and preferred use prompts a closer examination of the available evidence.

In this study, we critically reappraise the “renaissance of BD” [3]. We hypothesize that there is a methodological bias in the head-to-head comparison between BD and traditional vital parameters caused by imbalanced grouping and composite allocation.

## Methods

### Context and aims

The original publication compared BD with traditional ATLS parameters (HR, SBP, and GCS) for prediction of transfusion quantity. The study population was an observational cohort of 16,305 multitrauma patients derived from a German trauma register. Four shock classes (I-IV) were defined with modifications and numeric interpretations for HR, SBP, GCS, and BD (Table 1) [1, 2, 6].

**Table 1:** Interpretation of the ATLS shock classification of hypovolemic shock by Mutschler and colleagues using modifications and numeric interpretations [1, 2, 6].

	Class I	Class II	Class III	Class IV
Blood loss in %	< 15	15–30	30–40	> 40
Mental status	GCS = 15 (Slightly anxious or normal)	GCS = 15 (Mildly anxious)	GCS 12–14 (Anxious/confused)	GCS < 12 (Confused/lethargic)
Heart rate	< 100	100–120	120–140	> 140
Systolic blood pressure	≥ 110 (Normal)	≥ 100 (Normal)	< 100 (Decreased)	< 90 (Decreased)
Base deficit	≤ 2.0	> 2.0–6.0	> 6.0–10.0	> 10.0

*GCS* Glascow coma scale; Heart rate in beats per minute; Systolic blood pressure in mmHg; Base Deficit in mmol/L

Patients were categorized in shock classes corresponding to their HR, SBP, GCS, and BD upon arrival at the emergency department. Then, a composite ATLS score with classes I through IV was created corresponding to the shock classes of HR, SBP, and GCS. However, the majority of patients could not be placed in a single shock class because the shock class assigned to each individual vital parameter was different in 90.7% of cases. Thus, the composite ATLS score value was assigned to the shock class corresponding to the highest shock class amongst traditional vital parameters.

Finally, the quantity of transfused blood products during admission was collected. The composite ATLS-derived shock class was compared with the BD-derived shock class for transfusion quantity. Patients classified in shock class II, III and IV by BD received significantly more transfusions than patients classified by the composite ATLS score. The investigators concluded that “BD-based classification displayed a higher accuracy for discriminating the need for early blood products than the current ATLS classification of hypovolemic shock”.

We hypothesize that there is a systematic bias embedded in the methodological approach. The subjective cut-off selection causes imbalanced data groups, whilst composite allocation to the highest shock class causes complementary overestimation of patients’ shock class. This weakens the association between composite ATLS score and administered blood products. To illustrate, a young patient with stress and pain due to fractured bones may have a HR of 145, but otherwise normal vitals. A patient with a moderate traumatic brain injury may have a GCS of 11, but otherwise normal vitals. The grouping and composite allocation strategies will place both patients in shock class IV, although neither is likely to receive transfusion products.

To validate this hypothesis, we replicate findings of the original study without gathering any patient data. Instead, we used a simple computer simulation with generated data and apply the same methodology. Data simulation allows regulation of correlations between variables, enabling a focused investigation of potential methodological bias independent of signals from patient data. If all predictors (HR, SBP, GCS, and BD) are preset at equal correlation with the outcome (transfusion), no individual predictor should demonstrate a stronger association with the outcome. Therefore, if applying the methodology from the original study results in one predictor showing a significantly stronger association, it would indicate the presence of bias in the methodology. All simulations were performed using Python (v3.8, Jupyter Notebook) language for computing with a statistical suite of libraries. We adhered to a framework for design and reporting of simulation studies where applicable [5].

### Data simulation

We conducted a fully reproducible simulation study. First, we generated traditional ATLS vital parameters, BD, and transfusion outcome (units of packed red blood cells) in normal distributions based on average values and distributions as presented in the original cohort (supplemental material 1 and 2). Then, a copula was created to establish and regulate correlations of the joint multivariate distribution [7]. All correlations in the covariance matrix were initially set to 0.55 to simulate equal association between all variables. This correlation coefficient was selected as it represents the center of ‘moderate’ correlation and corresponds to a low number of patients in the same shock class [3, 8].

All distributions were truncated to physiological and realistic limits. For example, transfusion variable cannot be below zero and heart rate typically does not exceed 220. Moreover, GCS was rounded to integers and limited between 3 and 15. Its distribution was simulated trimodally with modes appearing at 3, 7, and 15, based on expected distributions from previous literature [9].

Traditional ATLS variables and BD were transformed into categorical shock class variables using the same thresholds as described in the original publication. The original publication classified a GCS of 15 into shock class I or II, corresponding to ‘normal’ or ‘mildly anxious’ mental status. We pragmatically, and randomly, redistributed half into class I and half into class II. Then, a composite ATLS score was created using the highest shock class allocation method from the original publication. As in the original publication, the BD and composite ATLS shock class were compared for transfusion quantity using a student’s t-test. Full code is available in supplemental material 3.

Finally, we changed the equivalent correlation to a weak correlation between BD and transfusion (rho = 0.3) and a strong correlation between HR, SBP, and GCS and transfusion (rho = 0.8) and repeated the same analysis. Moreover, to test whether the bias upholds independent of BD we compared HR shock class and highest allocation composite shock class of BD, SBP, and GCS using the same analysis with both correlation methods.

## Results

**Fig. 1:**
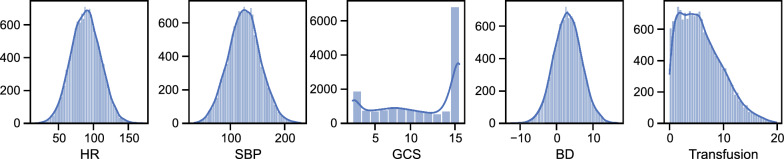
The distributions of simulated data. The y-axis represents value counts in the histograms. Transfusion in units packed red blood cells

Figure 1 shows distributions of simulated variables. Thirteen percent of patients were categorized in the same shock class for each traditional ATLS parameter. Supplemental material 4 shows the imbalanced distribution of cases across shock classes.

**Fig. 2:**
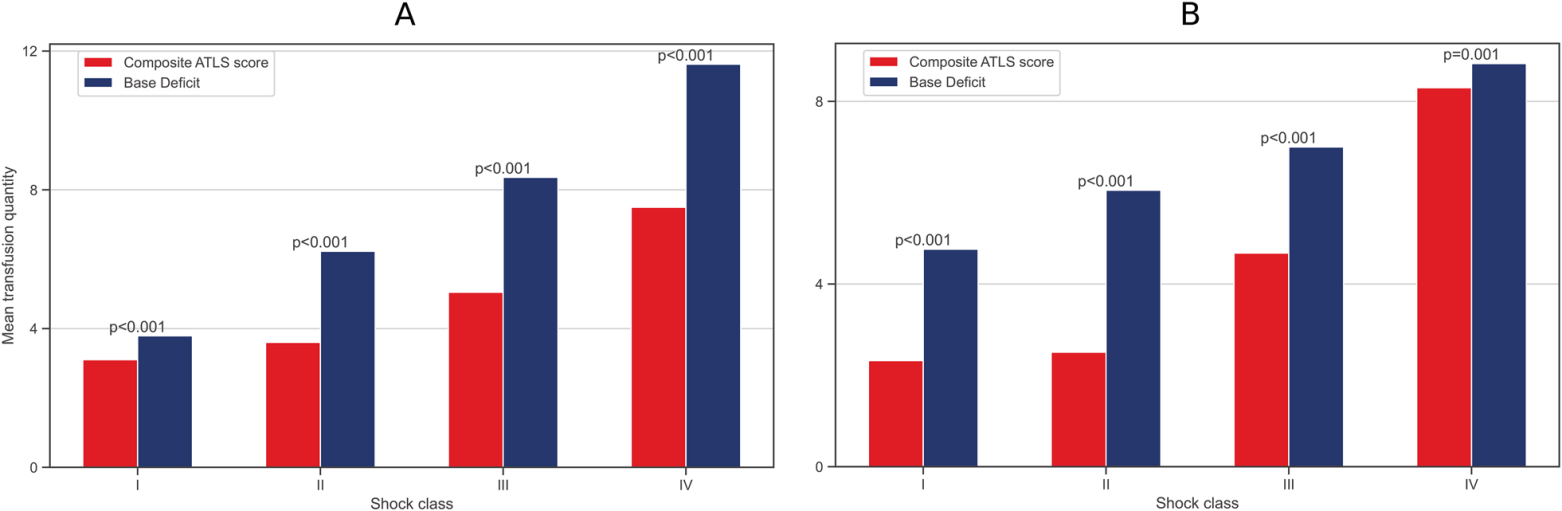
The transfusion quantity as a function of composite ATLS parameters and BD with equal correlations **A** and unequal correlations **B**. The post-processing Spearman correlation coefficient in equal correlations between transfusion and HR, SBP, GCS, and BD was 0.526, − 0.527, − 0.511, and 0.529, respectively. The composite ATLS score contains HR, SBP, and GCS. The post-processing Spearman correlation coefficient in unequal correlations between transfusion and HR, SBP, GCS, and BD was 0.791, − 0.787, − 0.765, and 0.272, respectively

Figure 2 shows the comparison of the composite ATLS score and BD for equal and unequal correlations. Note that BD seems to be superior for the identification of transfusion requirements even when its correlation with transfusion quantity is substantially lower. When comparing HR versus a composite of BD, SBP, and GCS, similar results occur in favor of HR (supplemental material 5).

## Discussion

In this simulation study we compare traditional ATLS vital parameters and BD for transfusion quantity using methodology described by a previous landmark publication. All variables were generated with an equal underlying correlation with transfusion quantity. First, despite these conditions, applying the original methodology produces a significant effect in favor of BD over traditional ATLS parameters, confirming the presence of a bias. Second, this effect persists when the correlation between transfusion and BD is set substantially lower than the correlation between transfusion and traditional ATLS parameters, emphasizing its magnitude. Last, the effect persists when comparing HR to a composite of BD, SBP, and GCS, indicating its independence from BD.

This study highlights how methodological choices may inadvertently influence results and conclusions of medical research. Allocating imbalanced data to higher composite shock classes leads to shock class overestimation and erroneous variable comparison. However, in clinical practice it is reasonable to be wary of outlier vital parameters in higher shock classes; initial overtreatment may be less hazardous than undertreatment.

Notably, despite being equivalent in root correlation, the categorized GCS group shows a consistent negative skew, whilst the other variables all show a positive skew. This can be attributed to grouping particularities. First, more than half of the original population has a GCS < 12, corresponding to shock class IV. Second, a GCS of 15 could be either class I or II depending on unspecified qualitative characteristics. Third, all parameters were measured at the emergency department, except for GCS when the patient was intubated at the scene, automatically classifying as shock class IV. These grouping-related factors consistently drive high shock class allocation for GCS without necessitating transfusion. Last, a low GCS in a patient with traumatic brain injury may not indicate ongoing systemic bleeding, potentially confounding the study results. Unfortunately, the original study did not clarify whether this specific subgroup of patients was excluded from the analysis.

Importantly, the relationship between BD and patient acuity is physiologically and epidemiologically sound. This study only identifies the methodological bias in the head-to-head comparison between BD and vital parameters in this specific landmark paper. Ultimately, resuscitation strategy should not be based on a singular ‘superior’ single-parameter, but rather on the integration of multiple parameters, context, repeated evaluation, and clinical judgement.

### Limitations

This study contains artificially generated data based on pre-specified averages, distributions, and correlations. In addition, it was truncated and post-processed to resemble natural data, marginally affecting correlations. We have responsibly selected, and accounted for, pre-specified context, but unexpected post-processing bias may still impact results. Moreover, in this study 13.92% of patients were classified in a joint shock class, compared to 9.3% in the original publication. Since imbalanced data drives allocation bias, we suspect that methodological bias may be even more pronounced in the original data. Last, this study was performed to investigate potential methodological bias, but is unable to determine the true underlying correlations between traditional ATLS parameters, BD, and transfusion quantity. We therefore look forward to a secondary analysis of original data or new data to resolve these matters.

## Conclusions

In this transparent and fully reproducible simulation we demonstrate the presence of a methodological bias in a landmark publication comparing ATLS parameters. The methodological bias causes favorable interpretation of BD’s value in shock classification compared to traditional parameters. Preferred use of BD over other vital parameters as a standard measure for acute hypovolemia cannot be justified using the analysis methods of the original study. Research with robust methods is needed to draw definitive conclusions on the “renaissance of BD”.

## Supplementary Information

Additional file 1

Additional file 2

Additional file 3

Additional file 4

Additional file 5

## Data Availability

The datasets generated and analyzed during the current study are available in the supplemental material section.

## Abbreviations

ATLS: Advanced trauma life support
BD: Base deficit
GCS: Glasgow coma scale
HR: Heart rate
SBP: Systolic blood pressure
