# Supplementary material for: Simulating the methodological bias in the ATLS classification of hypovolemic shock: a critical reappraisal of the base deficit renaissance
Source: Scand J Trauma Resusc Emerg Med. 2024 Oct 25;32:104. doi: 10.1186/s13049-024-01276-0 (PMC11515103; doi:10.1186/s13049-024-01276-0)
Supplement: Supplementary file 1 — Additional file 1 [file 13049_2024_1276_MOESM1_ESM.docx]

**Supplemental material 1**

All data taken from the original publication by Mutschler and colleagues.^1^ GCS, HR, and SBP are taken from figure 2 on the ED, as this is where the analysis was performed. The values for BD were taken as the centre of each range or +/-2 from the upper or lower limit. BDs standard deviation was estimated and rounded at 1.5, around 34.1% of the total range in class II and III. Only a singular transfusion quantity outcome was simulated to avoid complicating the demonstration of methodological bias. The values for the transfusion quantity outcome were taken from figure 1, corresponding to the amount of packed red blood cells transfused per class.

|  | Class I (7583) | Class II (5831) | Class III (1999) | Class IV (892) | Average |
| --- | --- | --- | --- | --- | --- |
| GCS | 14 (13-15) | 13 (6-15) | 10 (3-15) | 4 (3-12) | 12.6 (4.5) |
| HR | 86.3 (17.8) | 89.8 (20.3) | 95.9 (22.5) | 97.2 (32.4) | 89.3 (20.7) |
| SBP | 132.6 (26.3) | 124.6 (28.0) | 112.7 (30.7) | 94.8 (40.4) | 125.2 (30.0) |
| BD | 0 (1.5) | -4.0 (1.5) | -8.0 (1.5) | -12.0 (1.5) | -3.1 (3.8) |
| Transfusion | 1.2 (3.5) | 2.9 (5.6) | 5.7 (8.8) | 10.6 (4.9) | 2.9 (5.8) |

GCS Glasgow Coma Scale; HR heart rate; SBP systolic blood pressure; BD base deficit. Sample size is noted in brackets behind shock class. All data is represented as mean (standard deviation), except for GCS which is noted as median (interquartile range)

The code used to calculate the averages is attached in a portable document format (supplemental material 2)

**References**

1. Mutschler M, Nienaber U, Brockamp T, Wafaisade A, Fabian T, Paffrath T, Bouillon B, Maegele M; TraumaRegister DGU. Renaissance of base deficit for the initial assessment of trauma patients: a base deficit-based classification for hypovolemic shock developed on data from 16,305 patients derived from the TraumaRegister DGU®. Crit Care. 2013 Mar 6;17(2):R42. doi: 10.1186/cc12555. PMID: 23497602; PMCID: PMC3672480.
