# Supplementary material for: Simulating the methodological bias in the ATLS classification of hypovolemic shock: a critical reappraisal of the base deficit renaissance
Source: Scand J Trauma Resusc Emerg Med. 2024 Oct 25;32:104. doi: 10.1186/s13049-024-01276-0 (PMC11515103; doi:10.1186/s13049-024-01276-0)
Supplement: Supplementary file 2 — Additional file 2 [file 13049_2024_1276_MOESM2_ESM.pdf]

In [ ]:

```
import numpy as np
from scipy.stats import norm
```

In [ ]:

```
# Define the means and standard deviations for the population for SBP

# Data for each subgroup
subgroup_data = [
    {'mean': 132.6, 'std': 26.3, 'num_samples': 7583},
    {'mean': 124.6, 'std': 28.0, 'num_samples': 5831},
    {'mean': 112.7, 'std': 30.7, 'num_samples': 1999},
    {'mean': 94.8, 'std': 40.4, 'num_samples': 892}
]

# Calculate the weighted mean and standard deviation for the total population
total_samples = sum(subgroup['num_samples'] for subgroup in subgroup_data)
weighted_sum_mean = sum(subgroup['mean'] * subgroup['num_samples'] for subgroup in s
weighted_sum_std = sum((subgroup['std']**2 + subgroup['mean']**2) * subgroup['num_sa

population_mean = weighted_sum_mean / total_samples
population_std = np.sqrt(weighted_sum_std / total_samples - population_mean**2)

print("Population Mean:", population_mean)
print("Population Standard Deviation:", population_std)
```

In [ ]:

```
# Define the means and standard deviations for the population for HR

# Data for each subgroup
subgroup_data = [
    {'mean': 86.3, 'std': 17.8, 'num_samples': 7583},
    {'mean': 89.8, 'std': 20.3, 'num_samples': 5831},
    {'mean': 95.9, 'std': 22.5, 'num_samples': 1999},
    {'mean': 97.2, 'std': 32.4, 'num_samples': 892}
]

# Calculate the weighted mean and standard deviation for the total population
total_samples = sum(subgroup['num_samples'] for subgroup in subgroup_data)
weighted_sum_mean = sum(subgroup['mean'] * subgroup['num_samples'] for subgroup in s
weighted_sum_std = sum((subgroup['std']**2 + subgroup['mean']**2) * subgroup['num_sa

population_mean = weighted_sum_mean / total_samples
population_std = np.sqrt(weighted_sum_std / total_samples - population_mean**2)

print("Population Mean:", population_mean)
print("Population Standard Deviation:", population_std)
```

In [ ]:

```
# Define the means and standard deviations for the population for GCS

# Data for each subgroup
subgroup_data = [
    {'median': 14, 'iqr_range': (13, 15), 'num_samples': 7583},
    {'median': 13, 'iqr_range': (6, 15), 'num_samples': 5831},
    {'median': 10, 'iqr_range': (3, 15), 'num_samples': 1999},
    {'median': 4, 'iqr_range': (3, 12), 'num_samples': 892}
]

# Calculate the weighted median, estimated mean, and IQR for the total population
total_samples = sum(subgroup['num_samples'] for subgroup in subgroup_data)
weighted_median_values = []
```

```
weighted_mean_values = []
weighted_iqr_lower_values = []
weighted_iqr_upper_values = []

for subgroup in subgroup_data:
    subgroup_weight = subgroup['num_samples'] / total_samples
    weighted_median_values.append(subgroup['median'] * subgroup_weight)

    iqr_lower, iqr_upper = subgroup['iqr_range']
    weighted_iqr_lower_values.append(iqr_lower * subgroup_weight)
    weighted_iqr_upper_values.append(iqr_upper * subgroup_weight)

    # Calculate estimated mean using median and IQR
    estimated_mean = subgroup['median']
    weighted_mean_values.append(estimated_mean * subgroup_weight)

population_median = sum(weighted_median_values)
population_iqr_lower = sum(weighted_iqr_lower_values)
population_iqr_upper = sum(weighted_iqr_upper_values)
population_mean = sum(weighted_mean_values)

population_iqr = population_iqr_upper - population_iqr_lower

# Calculate estimated standard deviation (Assuming that the GCS follows a normal dis
estimated_stddev = (population_iqr / 1.35)

print("Population Median:", population_median)
print("Population Mean:", population_mean)
print("Population IQR:", population_iqr)
print("Estimated Standard Deviation:", estimated_stddev)
```

In [ ]:

```
# Define the means and standard deviations for the population for Transfusion of RBC

# Data for each subgroup
subgroup_data = [
    {'mean': 1.2, 'std': 3.5, 'num_samples': 7583},
    {'mean': 2.9, 'std': 5.6, 'num_samples': 5831},
    {'mean': 5.7, 'std': 8.8, 'num_samples': 1999},
    {'mean': 10.6, 'std': 4.9, 'num_samples': 892}
]

# Calculate the weighted mean and standard deviation for the total population
total_samples = sum(subgroup['num_samples'] for subgroup in subgroup_data)
weighted_sum_mean = sum(subgroup['mean'] * subgroup['num_samples'] for subgroup in s
weighted_sum_std = sum((subgroup['std']**2 + subgroup['mean']**2) * subgroup['num_sa

population_mean = weighted_sum_mean / total_samples
population_std = np.sqrt(weighted_sum_std / total_samples - population_mean**2)

print("Population Mean:", population_mean)
print("Population Standard Deviation:", population_std)
```

In [ ]:

```
# Define the means and standard deviations for the population for BE

# Data for each subgroup
subgroup_data = [
    {'mean': 0, 'std': 1.5, 'num_samples': 7583},
    {'mean': -4, 'std': 1.5, 'num_samples': 5831},
    {'mean': -8, 'std': 1.5, 'num_samples': 1999},
    {'mean': -12, 'std': 1.5, 'num_samples': 892}
]
```

```
# Calculate the weighted mean and standard deviation for the total population
total_samples = sum(subgroup['num_samples'] for subgroup in subgroup_data)
weighted_sum_mean = sum(subgroup['mean'] * subgroup['num_samples'] for subgroup in s
weighted_sum_std = sum((subgroup['std']**2 + subgroup['mean']**2) * subgroup['num_sa

population_mean = weighted_sum_mean / total_samples
population_std = np.sqrt(weighted_sum_std / total_samples - population_mean**2)

print("Population Mean:", population_mean)
print("Population Standard Deviation:", population_std)
```
