# Supplementary material for: Simulating the methodological bias in the ATLS classification of hypovolemic shock: a critical reappraisal of the base deficit renaissance
Source: Scand J Trauma Resusc Emerg Med. 2024 Oct 25;32:104. doi: 10.1186/s13049-024-01276-0 (PMC11515103; doi:10.1186/s13049-024-01276-0)
Supplement: Supplementary file 3 — Additional file 3 [file 13049_2024_1276_MOESM3_ESM.pdf]

In [ ]:

```python
import numpy as np
import pandas as pd
import matplotlib.pyplot as plt
import math
import seaborn as sns
import random
from scipy.stats import truncnorm, spearmanr, f_oneway, ttest_ind
from copulas.multivariate import GaussianMultivariate

sns.set_theme()
sns.set_style("ticks")

# Set the seed for reproducibility
seed = 42
np.random.seed(seed)
```

In [ ]:

```python
# Generating the distributions & parameters for the variables

n_cases = 16305

# Define the means and standard deviations (as taken from previous publication)
means = {
    "BD": 3.1,
    "SBP": 125.2,
    "HR": 89.3,
    "Transfusion": 2.9
}

stddevs = {
    "BD": 3.8,
    "SBP": 30.0,
    "HR": 20.7,
    "Transfusion": 5.8
}


limits = {
    "BD": (-15, 20),
    "SBP": (40, 250),
    "HR": (25, 220),
    "Transfusion": (0, 20),
}

normal_parameters = ["BD", "SBP", "HR", "Transfusion"]
dataset = pd.DataFrame()
for parameter in normal_parameters:

    dataset[parameter] = truncnorm.rvs(
        (limits[parameter][0] - means[parameter]) / stddevs[parameter],
        (limits[parameter][1] - means[parameter]) / stddevs[parameter],
        loc=means[parameter],
        scale=stddevs[parameter],
        size=n_cases
    )

# Simulating GCS by means of combining 3 normals into a trimodal distribution
gcs = {
    "GCS_1": pd.Series(np.random.normal(3, .5, 300)),
    "GCS_2": pd.Series(np.random.normal(7.5, 4, 5000)),
    "GCS_3": pd.Series(np.random.normal(15, .6, 3500)),
}
```

```
gcs_df = pd.concat([dist for dist in gcs.values()])

# Resampling into n_cases
gcs_df = gcs_df.sample(n_cases, replace=True).reset_index(drop=True)

#
dataset["GCS"] = gcs_df
parameters = ['HR', 'SBP', 'GCS', 'BD', 'Transfusion']
dataset = dataset[parameters]
```

In [ ]:

```
# Create copula to manage correlations
copula = GaussianMultivariate(random_state=seed)
copula.fit(dataset)
copula_params = copula.to_dict()
```

In [ ]:

```
# Set the correlation matrix used in the copula
# These variables can be tuned to create correlations as desired

# Create root correlation variables between vitals, BD, and transfusion
corr_root_vital = 0.55
corr_root_bd = 0.55

# Adjust all correlation to have the appropriate sign
corr_bd_sbp = -(corr_root_bd)
corr_bd_hr = (corr_root_bd)
corr_bd_gcs = -(corr_root_bd)
corr_bd_transfusion = (corr_root_bd)

corr_sbp_hr = -(corr_root_vital)
corr_sbp_gcs = (corr_root_vital)
corr_sbp_transfusion = -(corr_root_vital)

corr_hr_gcs = -(corr_root_vital)
corr_hr_transfusion = (corr_root_vital)

corr_gcs_transfusion = -(corr_root_vital)

# Create a correlation matrix based on these values
correlation_matrix = pd.DataFrame({
    "BD": [1, corr_bd_sbp, corr_bd_hr, corr_bd_gcs, corr_bd_transfusion],
    "SBP": [corr_bd_sbp, 1, corr_sbp_hr, corr_sbp_gcs, corr_sbp_transfusion],
    "HR": [corr_bd_hr, corr_sbp_hr, 1, corr_hr_gcs, corr_hr_transfusion],
    "GCS": [corr_bd_gcs, corr_sbp_gcs, corr_hr_gcs, 1, corr_gcs_transfusion],
    "Transfusion": [corr_bd_transfusion, corr_sbp_transfusion, corr_hr_transfusion,
}, index=["BD", "SBP", "HR", "GCS", "Transfusion"])

# Function to check whether the correlation matrix with input is valid
def _validate_correlation_matrix(correlation_matrix: np.ndarray) -> bool:
    # Check symmetry
    is_symmetric = np.allclose(correlation_matrix, correlation_matrix.T)

    # Check diagonal values
    diagonal_values = np.diagonal(correlation_matrix)
    all_diagonal_ones = np.allclose(diagonal_values, 1.0)

    # Check range
    is_in_range = np.all(np.logical_and(correlation_matrix >= -1, correlation_matrix

    # Check positive semidefiniteness
    eigenvalues, _ = np.linalg.eig(correlation_matrix)
    is_psd = np.all(eigenvalues >= 0)
```

```python
    # Print validation results
    print("Symmetry:", is_symmetric)
    print("Diagonal Values (All Ones):", all_diagonal_ones)
    print("Range [-1, 1]:", is_in_range)
    print("Positive Semidefinite:", is_psd, ", with eigenvalues:", eigenvalues)

    # Combine all checks to validate the correlation matrix
    is_valid_correlation_matrix = is_symmetric and all_diagonal_ones and is_in_range

    print("Is Valid Correlation Matrix:", is_valid_correlation_matrix)
    return is_valid_correlation_matrix

_validate_correlation_matrix(correlation_matrix=correlation_matrix)

# Print correlation matrix to check whether input is correct
correlation_matrix
```

In [ ]:

```python
# Apply selected correlation matrix to copula
simulation_copula_params = copula_params.copy()
simulation_copula_params["correlation"] = correlation_matrix
simulation_copula = GaussianMultivariate.from_dict(simulation_copula_params)
simulated_dataset = simulation_copula.sample(n_cases)
```

In [ ]:

```python
# Create a workable copy for the analysis
df = simulated_dataset.copy()
parameters = ['HR', 'SBP', 'GCS', 'BD', 'Transfusion']
df = df[parameters]
```

In [ ]:

```python
# Make sure GCS values are integers and limit them from 3 to 15
df["GCS"] = df["GCS"].round().astype(int)
df["GCS"] = np.clip(df["GCS"], 3, 15)

# Function to show distribution of correlated and postprocessed variables
def show_distributions(dataset: pd.DataFrame):

    # Determine the grid size: rows x columns
    fig, axes = plt.subplots(nrows=1, ncols=5, figsize=(12, 2))
    # Adjust the spacing between subplots on the x-axis
    plt.subplots_adjust(wspace=0.35)

    for i, column in enumerate(parameters):
        sns.histplot(dataset[column], kde=True, binwidth=1, ax=axes[i])
        axes[i].set(ylabel=None)
    fig.savefig(f'Distribution.eps', dpi=300, bbox_inches='tight')

    plt.show()

show_distributions(df[parameters])

# Show Spearman correlation matrix
df[parameters].corr(method="spearman")
```

In [ ]:

```python
# Grouping according to Mutschler bins

# BD according to ATLS (and as adapted by Mutschler and colleagues)
bd_bins = [-float('inf'), 2, 6, 10, float('inf')]
bd_labels = [1, 2, 3, 4]
```

```python
# HR according to ATLS (and as adapted by Mutschler and colleagues)
hr_bins = [-float('inf'), 100, 120, 140, float('inf')]
hr_labels = [1, 2, 3, 4]

# GCS according to Mutschler and colleagues (reappraisal of ATLS):
    # “Slightly anxious” and “mildly anxious” were GCS = 15 (class 1 or 2)
    # “anxious/confused” was GCS = 12–14 (class 3)
    # “confused/lethargic” was GCS < 12 (class 4)
gcs_bins = [-float('inf'), 11.99, 14.01, float('inf')]
gcs_labels = [4, 3, 1]

# According to Mutschler and colleagues (reappraisal of ATLS):
    # SBP of ≥110mmHg for group I,
    # ≥100mmHg for group II
    # <100mmHg for group III
    # <90mmHg for group IV
sbp_bins = [-float('inf'), 90, 100, 110, float('inf')]
sbp_labels = [4, 3, 2, 1]

# Create new categorical variables using defined cut-offs
df['BD ATLS'] = pd.cut(df['BD'], bins=bd_bins, labels=bd_labels).astype(int)
df['HR ATLS'] = pd.cut(df['HR'], bins=hr_bins, labels=hr_labels).astype(int)
df['GCS ATLS'] = pd.cut(df['GCS'], bins=gcs_bins, labels=gcs_labels).astype(int)
df['SBP ATLS'] = pd.cut(df['SBP'], bins=sbp_bins, labels=sbp_labels).astype(int)

# Adjust the GCS to contain 1 and 2 - randomly to set seed
df['GCS ATLS'] = np.where(df['GCS ATLS'] ==1,
                          np.random.choice([1,2],
                                           size=len(df)), df['GCS ATLS'])

# Count cases where the three variables have the same value
same_value_cases = df[["HR ATLS", "GCS ATLS", "SBP ATLS"]].apply(
    lambda row: row.nunique() == 1, axis=1).sum()

total_cases = len(df)

print(f"Total cases: {total_cases}")
print(f"Cases with same values: {same_value_cases}")
print(f"Percentage: {same_value_cases / total_cases * 100:.2f}%")

# Do GCS groups approximately represent data in original publication table 4?
print("GCS I-II pct of total", ((df["GCS ATLS"] == 1) | (df["GCS ATLS"] == 2)).sum()
print("GCS III pct of total", (df["GCS ATLS"] == 3).sum() / len(df))
print("GCS IV pct of total", (df["GCS ATLS"] == 4).sum() / len(df))
```

In [ ]:

```python
# Histograms for ATLS shock class I-IV per variable

# Create subplots for histograms
fig, axes = plt.subplots(nrows=1, ncols=4, figsize=(15, 4))

# Variables to plot
variables = ['HR ATLS', 'SBP ATLS', 'GCS ATLS', 'BD ATLS']

# Create histograms for each variable
for i, ax in enumerate(axes.flatten()):
    variable = variables[i]
    ax.hist(df[variable], bins=[1, 2, 3, 4, 5], align='left', rwidth=0.8, alpha=0.7)
    ax.set_title(f'{variable}')
    ax.set_xlabel('Shock class')
    ax.set_ylabel(None)
    ax.set_xticks([1, 2, 3, 4])
    ax.set_xticklabels(['I', 'II', 'III', 'IV'])
```

```python
# Remove the last subplot
if len(variables) < len(axes.flatten()):
    fig.delaxes(axes.flatten()[-1])

# Adjust layout, save, and display plots
fig.savefig(f'Histograms.eps', dpi=300, bbox_inches='tight')
plt.tight_layout()
plt.show()
```

In [ ]:

```python
# Create variable for allocation to highest ATLS shock class
df['ATLS highest'] = df[['HR ATLS', 'GCS ATLS', 'SBP ATLS']].max(axis=1)
```

In [ ]:

```python
# Compare composite traditional ATLS parameters with BD
def plot_shock_classes(df, parameters, parameter_names, ax):
    parameter_indexes = [1, 2, 3, 4]
    bar_width = 0.3
    index = range(4)

    cat_df = df.copy()

    means = []

    for parameter in parameters:
        means.append(cat_df.groupby(parameter)["Transfusion"].mean().reindex(
            parameter_indexes, fill_value=None).sort_index())

    # Calculate p-values within each parameter index
    p_values = []
    for cat_index in parameter_indexes:
        category_data = [cat_df[cat_df[parameter] == cat_index]['Transfusion'] for p
        f_statistic, p_value = ttest_ind(*category_data)
        p_values.append(p_value)

     # Define custom bar colors to match "Critical Care" colors
    bar_colors = ["#D9030F", "#0D2A6E"]

    # Plot the bar charts
    for i in range(len(parameters)):
        x_positions = [x + i * bar_width - (bar_width * 0.5) for x in index]  # Adju
        bars = ax.bar(x_positions, means[i], bar_width, label=parameter_names[i], co

    # Annotate one p-value per category index
    for j, p_value in enumerate(p_values):
        x_location = index[j]  # Center of the x-axis tick
        highest_bar = max([means[i].iloc[j] for i in range(len(parameters))])
        if p_value < 0.001:
            annotation_text = f'p<0.001'
        else:
            annotation_text = f'p={p_value:.3f}'
        ax.annotate(annotation_text, xy=(x_location, highest_bar + 0.02),
                    ha='center', va='bottom')

    # Setting the title, labels, and legend
    ax.set_title('Mean transfusion quantity per shock class')
    ax.set_xlabel('Shock class')
    ax.set_ylabel('Mean transfusion quantity')
    ax.set_yticks(ax.get_yticks()[::2])
    ax.set_xticks([x for x in index])  # Position the parameter label at the center
    ax.set_xticklabels(['I', 'II', 'III', 'IV'])
    ax.legend()

# Create a list of categories and their names
```

```
parameters = ["ATLS highest", "BD ATLS"]
parameter_names = ["Composite ATLS score", "Base Deficit"]

# Create the figure and axis
fig, ax = plt.subplots(figsize=(10, 6))

# Call the function to plot the parameters
plot_shock_classes(
    df=df,
    parameters=parameters,
    parameter_names=parameter_names,
    ax=ax
)

# Save, show grid, show legend
plt.grid(axis='y')
plt.legend(loc='upper right', bbox_to_anchor=(0.32, 1.0))
fig.savefig('Transfusion per shock class.eps', dpi=300, bbox_inches='tight')
plt.show()
```
