# Supplementary figures and images for: Simulating the methodological bias in the ATLS classification of hypovolemic shock: a critical reappraisal of the base deficit renaissance
Source: Scand J Trauma Resusc Emerg Med. 2024 Oct 25;32:104. doi: 10.1186/s13049-024-01276-0 (PMC11515103; doi:10.1186/s13049-024-01276-0)

HR ATLS

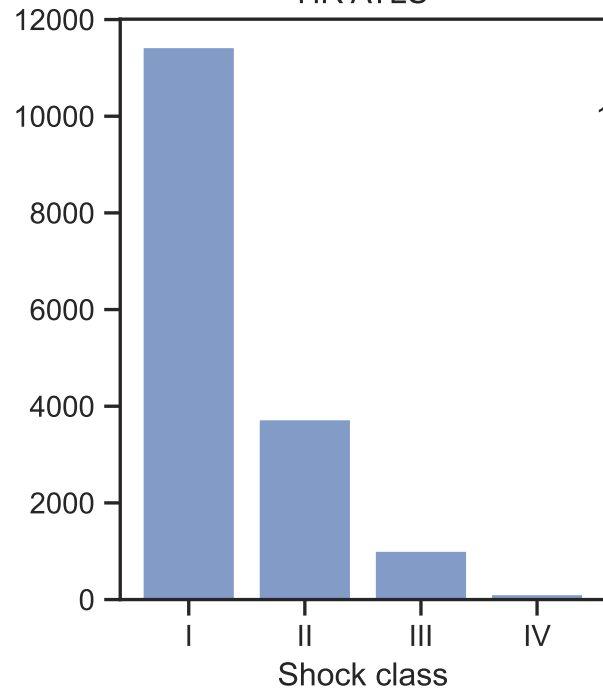

SBP ATLS

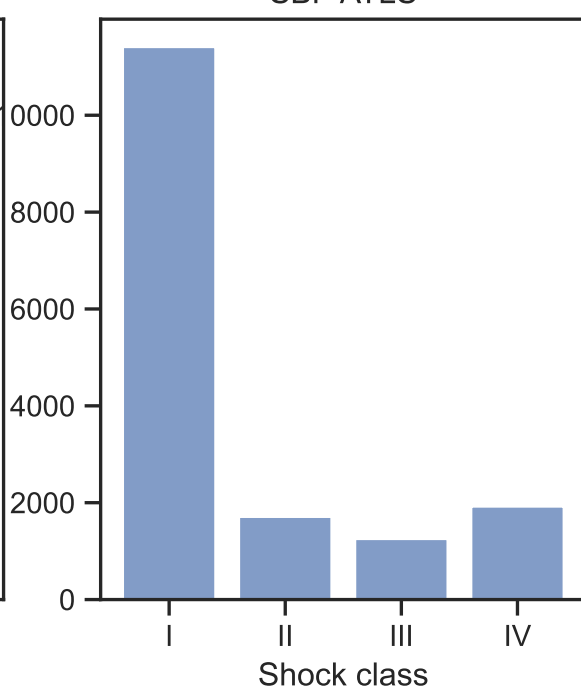

GCS ATLS

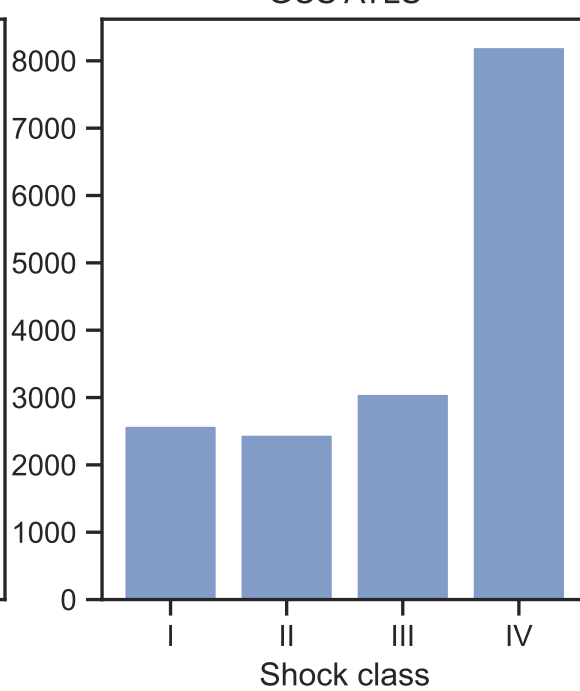

BD ATLS

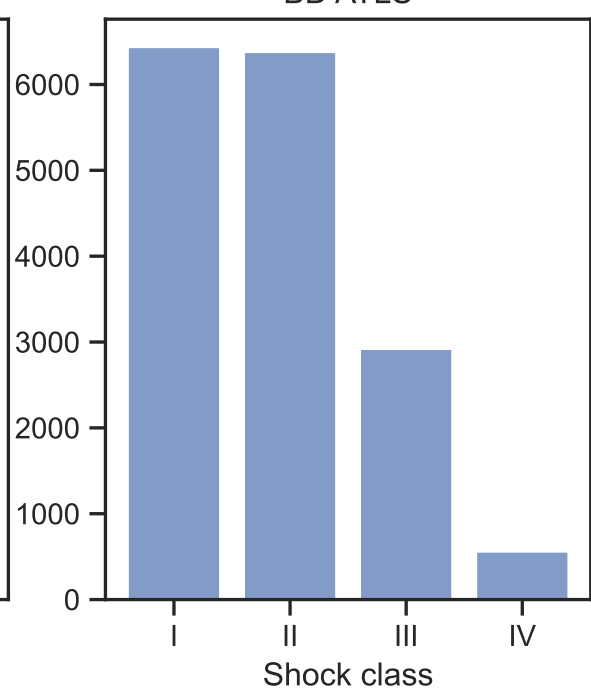

Supplement: Supplementary file 4 — Additional file 4 [file 13049_2024_1276_MOESM4_ESM.pdf]
