# Supplementary material for: Simulating the methodological bias in the ATLS classification of hypovolemic shock: a critical reappraisal of the base deficit renaissance
Source: Scand J Trauma Resusc Emerg Med. 2024 Oct 25;32:104. doi: 10.1186/s13049-024-01276-0 (PMC11515103; doi:10.1186/s13049-024-01276-0)
Supplement: Supplementary file 5 — Additional file 5 [file 13049_2024_1276_MOESM5_ESM.docx]

**Supplemental Material 5**

In this simulation the HR shock class is compared to the highest composite allocation shock class of BD, SBP, and GCS for transfusion quantity.

The figure below shows the comparison of HR to composite BD, SBP, and GCS with all variables having equal underlying correlations (rho=0.55)


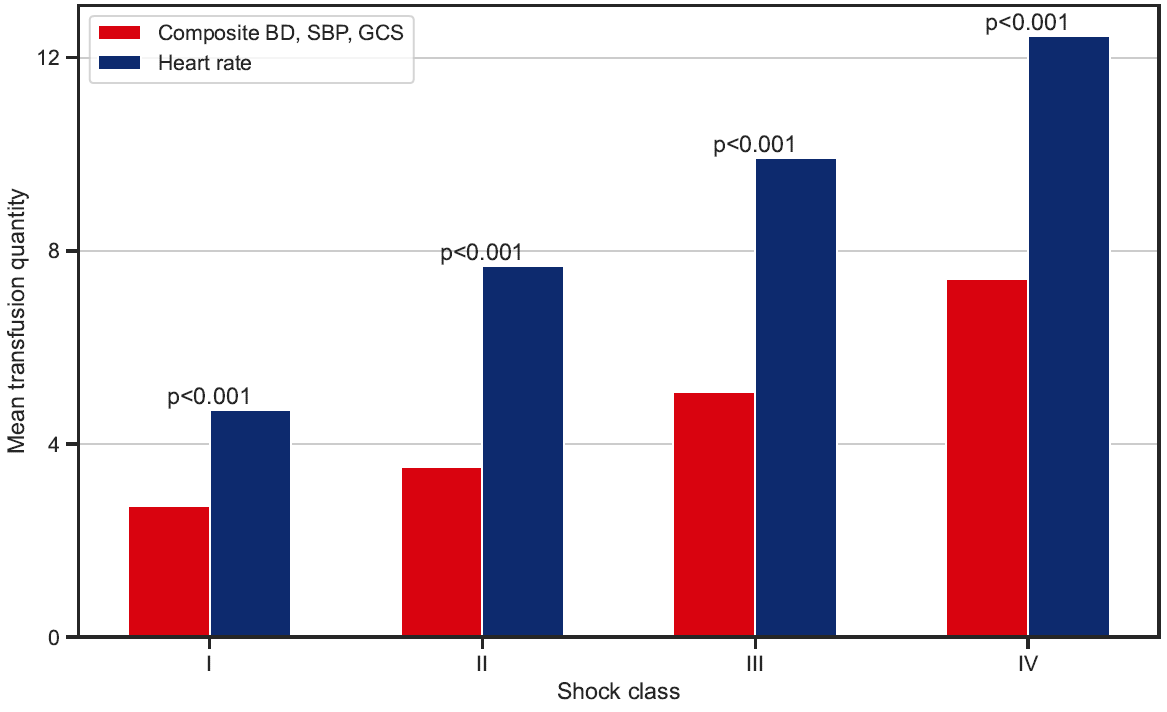


The figure below shows the comparison of HR to composite BD, SBP, and GCS for transfusion quantity with the correlation between transfusion and HR set to rho=0.30 and the correlation between composite BD, SBP, and GCS set to rho=0.80.


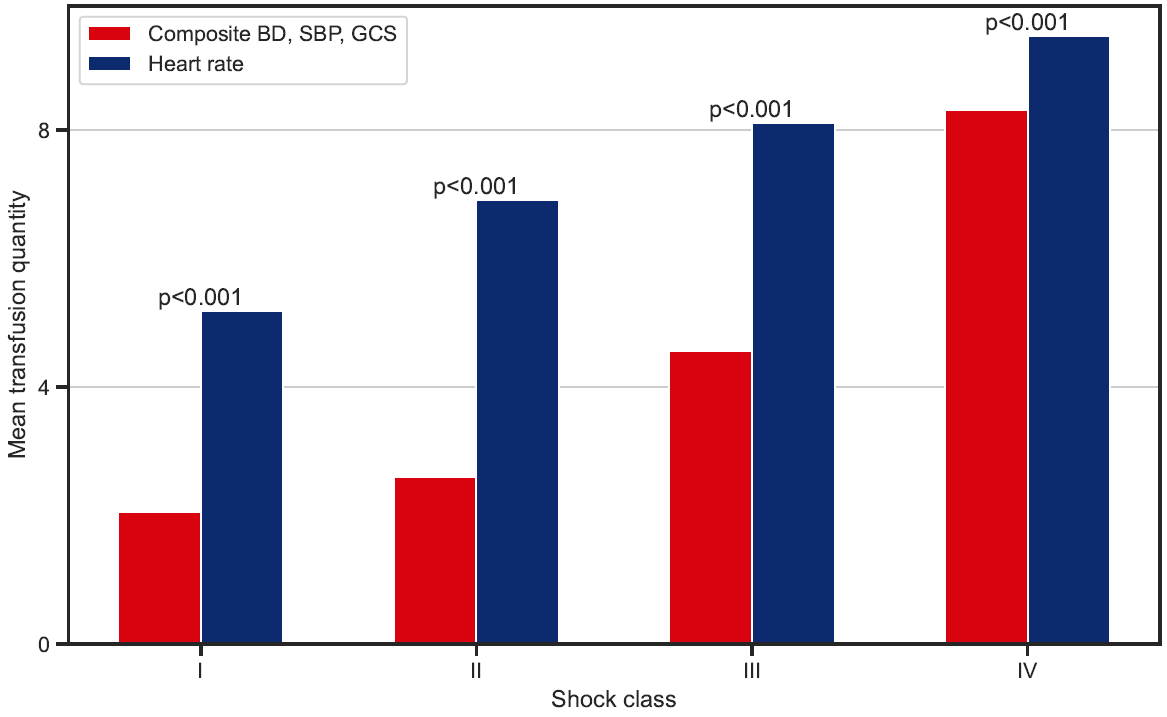


All results significantly favor HR despite its correlation with transfusion quantity being equal or substantially worse than the correlation between the composite and transfusion quantity.
